# Comparison of Radical Nephroureterectomy and Partial Ureterectomy for the Treatment of Upper Tract Urothelial Carcinoma

**DOI:** 10.1155/2018/2793172

**Published:** 2018-04-26

**Authors:** Jianzhong Zhang, Feiya Yang, Mingshuai Wang, Yinong Niu, Weicheng Chen, Nianzeng Xing

**Affiliations:** Department of Urology, Beijing Chaoyang Hospital, Capital Medical University, Beijing 100020, China

## Abstract

This study aimed to compare the oncological and renal outcomes of partial ureterectomy (PU) versus radical nephroureterectomy (RNU) for upper tract urothelial carcinoma (UTUC). UTUC patients' clinical information was reviewed, and progression-free survival (PFS), overall survival (OS), and kidney function were collected. The mean follow-up period was 59 (6–135) months in the RNU group and 34.5 (5–135) months in the PU group. The mean operation time in the PU group was 141 (64–340) min, which is significantly shorter than the RNU group (*P* < 0.01). Regarding kidney function at one year or two years after operation, the PU group had significantly improved mean estimated glomerular filtration rate (eGFR) levels and a remarkably decreased constitution of patients with chronic kidney disease (CKD) III or higher group (*P* < 0.05). There was no significant difference in PFS and OS between the RNU group and the PU group (*P* > 0.05). Multifactor Cox regression analysis indicated that age and the preoperative CKD stages were independent risk factors for poor kidney functions of UTUC patients. Compared to patients in RNU group, patients in PU have no significant difference in survival time but have shorter operation time, shorter hospital stay, and improved kidney functions.

## 1. Introduction

Upper tract urothelial carcinoma (UTUC) is a rare malignancy, accounting for 5% of all urothelial carcinoma and 10% of renal tumors [1, 2]. Generally, UTUC is considered to possess higher grade and stage, and the prognosis of UTUC is poorer compared with kidney cancer or bladder cancer. As a result, the recommended treatment is radical nephroureterectomy (RNU) with removal of the ipsilateral bladder cuff [3]. Historically, this radical procedure was performed through an open approach. But minimally invasive surgery, such as laparoscopic or robotic-assistant approach, has become popular in recent years [4, 5]. Long-term follow-up of UTUC patients who were treated by radical surgery suggests that open approach has oncologic equivalence to minimally invasive surgery without significant differences in overall survival and progression-free survival [6, 7]. Nevertheless, despite the approach of radical surgery, the prognosis of patients with advanced UTUC (stage T3-4) is poor. The 5-year survival of which ranges from 12% to 54% [8]. More importantly, radical surgery dramatically increases the risk of cardiovascular events due to the decline of renal function during the follow-up of patients after operation [9, 10]. UTUC consists of carcinoma of the renal pelvis and the carcinoma of the ureter. During the past decades, the incidence of ureter cancer increased slightly from 0.69/10^4^ per year to 0.73/10^4^ per year [11]. It is worth noting that 70% of ureter tumors occurred at the distal ureter, 25% at middle ureter, and 5% at the proximal ureter [12, 13]. In addition, about 55% to 75% of ureter cancer cases were found to be relatively better differentiated and lower in tumor staging compared with the malignancies of the renal pelvis [13]. In that case, it is extremely possible that a large proportion of ureter tumors might be overtreated by RNU. As a result, for UTUC patients with low risk of invasion, excision of the kidney, the full length of the ureter, and the bladder cuff during a nephroureterectomy remain controversial.

It has become increasingly recognized that, in addition to the radical surgery, a partial ureterectomy (PU) could be used for specific UTUC patients with low risk of aggressiveness. PU could save more kidney tissue compared with radical surgery and improve the patient's renal function. Therefore, PU could reduce the risk of cardiovascular death caused by chronic kidney disease [10]. Importantly, the adverse patient and tumor characteristics might be different between PU and RNU, but the updated evidence suggests that PU and RNU have similar oncological outcomes [14]. The recurrence-free survival and intravesical recurrence-free survival were similar between PU and RNU. However, to date, few reports could be found to compare the prognosis of UTUC patients who underwent radical surgery versus PU in the Chinese population. Hence, in this study, we aim to compare the oncological and renal outcomes of PU versus RNU for UTUC and to provide evidence for the decision-making for the surgical management of UTUC.

## 2. Patients and Methods

### 2.1. Study Design and Setting

In this retrospective cohort study, UTUC patients who underwent RNU or PU at the Department of Urology, Beijing Chaoyang Hospital, Medical Capital University, were included. Patients' clinical information was reviewed, and the follow-up parameters include the progression-free survival (PFS) and the overall survival (OS), as well as the kidney functions. This study was approved by the Institutional Review Board of the Beijing Chaoyang Hospital, Medical Capital University. Patients' informed consent for both the collection of hospital medical records and follow-up was obtained.

### 2.2. Participants

Between June 2005 and June 2016, UTUC patients who underwent RNU or PU at the Department of Urology, Chaoyang Hospital, Beijing Capital University, were included. The included patients were all pathologically diagnosed with primary urothelial carcinoma located at the upper tract of the ureter and underwent surgical management. Patients with distal metastasis, immune deficiency, and UTUC occurring at the transplanted urinary system or patients with severe cardiovascular disease, hepatic disease, or pulmonary disease that could not tolerate the operation were excluded from this study. As a result, complete data on age, gender, smoking status, Eastern Cooperative Oncology Group (ECOG) score, tumor stage, operation duration, and estimated glomerular filtration rate (eGFR), as well as chronic kidney disease (CKD) stage were available for all the included patients that met the inclusion criteria.

According to the type of surgery, all the included patients were divided to either a RNU group or a PU group. The patient's treatment was decided by their clinician in consultation with the patient. The patients receiving PU included low-risk patients (such as those with a sole tumor, diameter < 2 cm, low grade, with no invasion on radiographic image [15]), older patients who may be intolerant of RNU surgery, high-risk patients with renal dysfunction or a solitary kidney, patients who were predicted to have renal insufficiency after nephrectomy, and patients who were predicted to receive postoperative chemotherapy. Some patients lacking cytology or ureteroscopy biopsies underwent PU and pathology indicated high grade.

### 2.3. Pathological Evaluation

Pathological verification was obtained via examination by dedicated genitourinary pathologists at Chaoyang Hospital, Beijing Capital Medical University. Tumor grading was evaluated based on the 1998 World Health Organization/International Society of Urologic Pathology WHO/ISUP consensus [16]. Tumors stages were assessed according to the updated TNM classification by the American Joint Committee on Cancer/International Union Against Cancer (AJCC/UICC) [17].

### 2.4. Variables

Hospital medical records of all the included patients were reviewed to obtain the presentation of the disease, the pathological verification, the indication for treatment, and clinical outcomes, such as postoperative complications, kidney function, the recurrence of tumors, disease progression, and survival status. Disease progression was defined as the upstaging of clinical or radiological features or subsequent pathological upgrading or upstaging in patients who underwent nephroureterectomy. The PFS in this study is defined as the length of time after the PU or RNU that a patient lives with the disease without disease progression. The kidney event was considered if patients who were not at baseline CKD III had progressed to CKD III after surgical treatment or patients with CKD III staging further worsened during the follow-up.

### 2.5. Follow-Up

The initial follow-up of patients who underwent RNU or PU included cystoscopy or ureteroscopy surveillance at 3 months. In the first year after operation, patients were followed up every 3 months. In the second year, patients were followed up every 6 months. From the third year after the surgery, patients were followed up once a year. The length of further follow-up intervals was determined according to the presence of tumor recurrence. The contents of follow-ups included creatinine, imaging, and cystoscopy. The oncology outcome was evaluated by intravenous urography (IVU) or computed tomography [8] urography. The renal outcome was evaluated by serum creatinine at 3 months, 12 months, and 24 months after the surgery.

### 2.6. Statistical Methods

Statistical analysis was performed using SPSS version 22.0 (IBM, New York, NY, USA). Continuous variables that conform to the normal distribution were expressed as means ± SD, and the median variable that did not conform to the normal distribution was expressed by median [18]. Continuous variables conforming to the normal distribution were tested using the *t*-test, and the Mann–Whitney *U* test was performed using continuous variables that did not conform to the normal distribution. Classification variables were analyzed by chi-square test or Fisher exact test, PFS, OS, kidney events using K-M curves, and log-rank tests. Age, sex, preoperative CKD stage, and operation were analyzed by multivariate Cox regression model.

## 3. Results

A total of 157 patients were included, including 109 cases who underwent RNU and 48 cases who underwent PU. The median follow-up time was 59.5 months (6–135) in the RNU group and 34.5 months (5–135) in the PU group.  Table 1 illustrates the descriptive characteristics of the included patients who were treated with RNU or PU. The median [18] age was 67 (43–86) in the RNU group and was 75 (57–83) in the PU group, which was significantly different (*P* = 0.011). The sex constitution was comparable between the RNU group (52/109, 47.71%) and the PU group (23/48, 47.92%, *P* = 0.981). Importantly, the constitution of tumor stage had no significant difference (*P* = 0.444). For localized tumors (Tis-T1), there were 28 (25.69%) in the RNU group and 15 (31.25%) in the PU group. For locally advanced tumors (T2-T3), there were 80 (73.39%) and 32 (66.67%) in the RNU group and the PU group, respectively. Therefore, the constitution of tumor grade between the two groups had no statistical difference (*P* = 0.392). Moreover, the kidney function, one of the primary outcomes for this study, was statistically compared between the RNU and PU groups based on the eGFR value (*P* = 0.878) and CKD stage (*P* = 0.912). Overall, the baseline characteristics between the RNU and the PU groups had no significant difference regarding the comparison of the main outcomes applied in this study.

Table 2 summarized the main features of surgical procedures in both the RNU group and the PU group. Overall, laparoscopic operation was more frequently applied in the RNU group compared with PU group (*P* < 0.001). In addition, the requirement for blood transfusion was similar between the RNU group (7/109, 6.4%) and the PU group (2/48, 4.2%, *P* = 0.723). To evaluate the technical difficulty and perioperative recovery, we further compared the operation time and the length of stay at hospital for UTUC patients included in this study. Notably, the operation time of PU (141 mins, 64–340 mins) was sharply reduced compared with the RNU group (288 mins, 110–455 mins, *P* < 0.001). More importantly, the length of stay at hospital for UTUC patients who underwent PU was also dramatically reduced to 17.5 days (5–37 days), compared to patients who underwent the RNU (21 days, 8–67 days, *P* = 0.045, Table 2).

**Figure 1:**
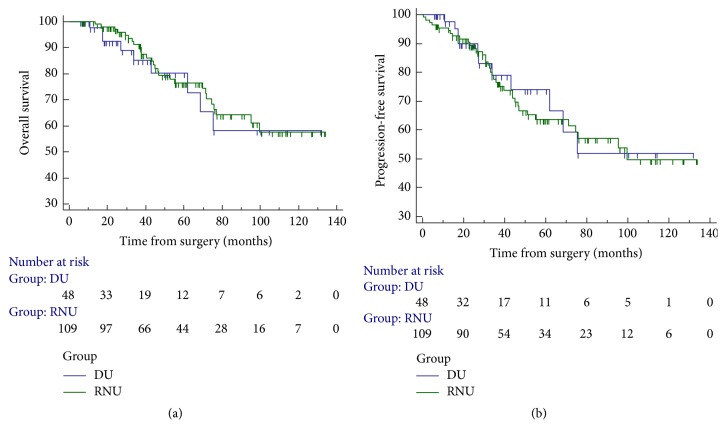
Overall survival (OS) and progression-free survival (PFS) of upper tract urothelial cell carcinoma (UTUC) patients. (a) Overall survival (OS) of upper tract urothelial cell carcinoma (UTUC) patients who underwent radical nephroureterectomy (RNU) or partial ureterectomy (PU) (log-rank test, *P* = 0.694). (b) Progression-free survival (PFS) of upper tract urothelial cell carcinoma (UTUC) patients who underwent radical nephroureterectomy (RNU) or partial ureterectomy (PU) (log-rank test *P* = 0.778).

Next, we sought to explore the differences in oncological outcomes between the RNU group and the PU group. Median follow-up time was 59.5 months (6–135) in the RNU group and 34.5 months (5–135) in the PU group. Nevertheless, there was no significant difference in the overall survival (OS) of UTUC patients who underwent RNU or PU (log-rank test, *P* = 0.694). Moreover, the progression-free survival (PFS) of UTUC patients who underwent RNU or PU was statistically similar as well (log-rank test *P* = 0.778, Figure 1).

**Figure 2:**
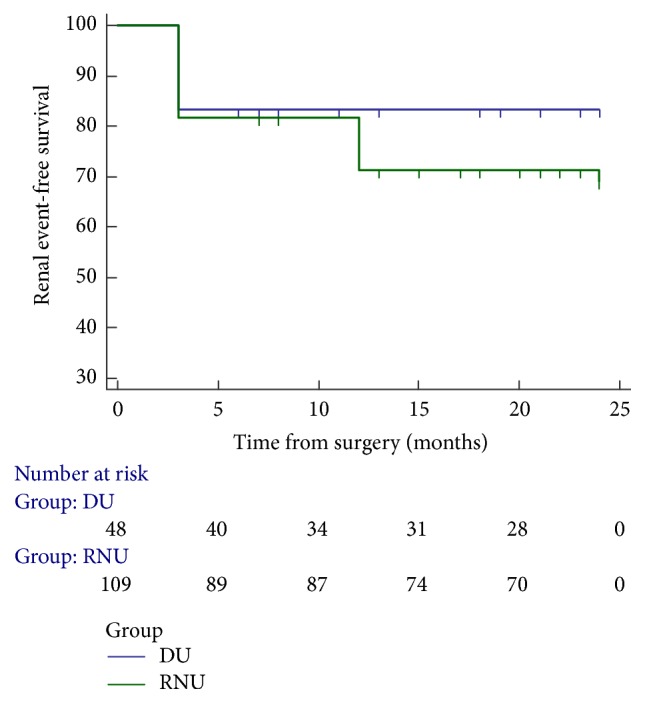
Event-free survival of upper tract urothelial cell carcinoma (UTUC) patients. Event-free survival of upper tract urothelial cell carcinoma (UTUC) patients who underwent radical nephroureterectomy (RNU) or partial ureterectomy (PU) (log-rank test *P* = 0.131).

There were no significant differences in the odds of CKD III or higher and the mean values of eGFR between the RNU group and the PU group at three months after the operation (*P* > 0.05, Table 3). However, during the follow-up of UTUC patients after 12 months after surgery, the renal functions in the PU group were significantly improved compared with the RNU group. After 24 months after surgery, during follow-up, the eGFR of the PU group (61.56 ± 25.68) was sharply increased compared with the eGFR of the RNU group (50.01 ± 20.36, *P* = 0.010). In addition, the constitution of patients with CKD III or higher in the RNU group was 53.21% (58/109), while in the PU group it was 25% (12/48, *P* = 0.008, Table 3). Furthermore, during the follow-up, event-free survival of UTUC patients who underwent PU was potentially improved compared with the RNU group according to Kaplan-Meier Curve (Figure 2, *P* = 0.131). To explore the critical factors that affect the survival of UTUC patients, the multivariate Cox regression analysis was performed including potential risk factors, such as age, sex, CKD stage, and surgery. As a result, age and CKD stage were demonstrated to significantly contribute to the survival of UTUC patients in this study (Table 4, *P* < 0.05).

## 4. Discussion

Radical organ-sparing surgery can be implemented for a number of malignant diseases with specific indications, such as breast cancer, kidney cancer, or penile cancer [19–21]. The objective of applying this organ-sparing method is to improve the quality of life as well as the functional outcome. However, malignancy that occurs at the urinary tract epithelia is a regional defect, as the whole urinary tract epithelium is considered to be at risk. In this case, organ-sparing surgery was an exception and is not recommended. Typically, for patients who were not able to tolerate RNU or had imperative indications, such as renal insufficiency, solitary kidneys, or bilateral tumor, PU, not RNU, should be considered as treatment for UTUC [22]. Based on the practice of these cases who underwent PU, the effective oncologic control of PU is highlighted recently [23]. In keeping with this notion, in the current retrospective studies comparing RNU and PU for the treatment of UTUC, the imperative indications constitute the majority of the criteria for PU [24]. Therefore, clinical studies using the updated consensus for the selection of PU patients are rare. In addition, there is an ongoing debate about the risk factors that affect the recurrence for patients with UTUC who have underwent surgical management [18]. To fill this gap and provide updated evidence for the decision-making of UTUC treatment, we performed this retrospective study to further evaluate the role of PU.

In this study, among the 157 UTUC cases included in this study, 109 patients underwent RNU and were assigned to the RNU group, while 48 cases were in the PU group. The mean operation time in the PU group was significantly shorter than the RNU group (*P* < 0.01). Regarding the kidney function at 1 year or 2 years after operation, the PU group had significantly higher mean eGFR levels than the RNU group and a remarkably decreased constitution of patients with CKD III or higher stage (*P* < 0.05). Importantly, there was no significant difference in PFS and OS between the RNU group and the PU group (*P* > 0.05). Multifactor Cox regression analysis indicated that age and the preoperative CKD stages were independent risk factors for poor kidney functions of UTUC patients. Overall, UTUC patients treated by PU and RNU have no significant difference in survival time, but PU patients have shorter operation time and hospital stay, as well as improved long-term outcomes of kidney functions.

Although the surgical management of UTUC has been extensively reviewed previously, the prognosis of PU remains controversial [25–27]. Seisen et al. performed a multi-institutional study with 304 UTUC patients who underwent RNU, distal ureterectomy, or endoscopic surgery [28]. There were no significant differences between RNU and distal ureterectomy in the 5-year local recurrence-free survival, rates of overall survival, and cancer specific survival [28]. Furthermore, Seisen et al. systematically reviewed the current literature comparing the oncologic outcomes of PU versus RNU for patients with UTUC and found that, for low-grade and noninvasive UTUC tumors, the oncologic outcomes were similar between PU and RNU whenever using ureteroscopic or percutaneous management [29]. Furthermore, for patients with high-grade and invasive UTUC, segmental ureterectomy was feasible as well [29]. In keeping with that, the indications for PU might be extended to selected cases of high-grade or invasive UTUC tumors. As the top-level clinical evidence, another two papers include a systematic review and a meta-analysis comparing PU with RNU [27, 30]. As a result, these studies collectively suggest that kidney-sparing management had similar effects on prognosis on UTUC as the standard nephroureterectomy. In this study, our data collectively demonstrated that there were no significant differences in the OS and PFS of UTUC patients who underwent RNU or PU.

Interestingly, there were no significant differences in the renal function between the RNU group and the PU group at three months after the operation. In accordance with these results, Singla et al. included 193 patients with UTUC who underwent RNU or PU over a median follow-up of 25.9 months and reported that rates of new-onset CKD or worsening of CKD stage were similar in patients treated with PU and RNU [31]. However, in our study, the renal functions in the PU group were significantly improved compared with the RNU group at one year or two years after the surgery. These findings imply an opportunity for better quality of life, if patients underwent the kidney-sparing operation. This phenomenon is related to surgical relieving of obstruction in the PU group, which directly leads to the improvement of renal function. More importantly, the preservation of the kidney unit plays an important role for the improvement of life expectancy.

Regarding the operation time, it was dramatically reduced in the PU group (141 mins, 64–340 mins) compared with the RNU group (288 mins, 110–455 mins). This is reasonable because the technical procedures for RNU are more complicated than the PU group, which do not need the excision of the kidney. In keeping with that, the trauma brought to the patients by PU is sharply decreased compared with the RNU treatment. In this case, the small resection range, short operative time, and low complication rate contribute to the decline of trauma in the PU treatment. Therefore, the hospital stay as well as the cost for patients in the PU group are significantly reduced.

A limitation of our present study is the study design. Since UTUC is a relatively rare disease, we applied the retrospective cohort study to elucidate the potential role of RNU or PU in the treatment of UTUC. In addition to the retrospective design, the single institution study and limited sample size are also the limitations of the current study. Moreover, these results should be interpreted with caution for lack of subgroup analysis according to stage and grade of UTUC. Current European Urology guidelines suggest conservative treatment for low-risk patients, but in this study some high-risk patients were included in the PU group. Further perspective random controlled trails with a large sample size are needed to confirm the role of PU in the treatment of UTUC.

## 5. Conclusions

Our study suggests that, compared with cases who underwent RNU, UTUC patients treated by PU have no significant difference in survival time but have shorter operation time and hospital stay, as well as improved long-term outcomes of kidney functions. For specific UTUC patients, PU shows more favorable benefits compared with RNU treatment. However, further perspective random controlled trails with a large sample size are needed to confirm the recommendation.

## Figures and Tables

**Table 1:** Baseline characteristics of patients who underwent RNU or PU.

	RNU (*N* = 109)	PU (*N* = 48)	*P* value
Age	67 (43–86)	75 (57–83)	0.011
Male	52 (47.71%)	23 (47.92%)	0.981
ECOG			<0.001
0	109 (100%)	39 (81.25%)	
1	0 (0%)	9 (18.75%)	
Pathological tumor stage			0.444
Tis-T1	28 (25.69%)	15 (31.25%)	
T2-T3	80 (73.39%)	32 (66.67%)	
Tumor grade			0.392
Low	37 (33.94%)	21 (43.75%)	
High	71 (65.14%)	26 (54.17%)	
Tumor multifocality	15 (13.8%)	5 (10.4%)	0.562
Adjuvant chemotherapy	0	0	
Lymph node dissection	0 (0%)	2 (4.2%)	0.092
Carcinoma in situ	3 (2.8%)	2 (4.2%)	0.642
eGFR	65.1 ± 26.9	65.8 ± 25.45	0.878
CKD stage III or higher	51 (46.79%)	22 (45.83%)	0.912
Follow-up	59 (6–135)	34.5 (5–135)	0.002

RNU: radical nephroureterectomy; PU: partial ureterectomy; ECOG: Eastern Cooperative Oncology Group; eGFR: estimated glomerular filtration rate; CKD: chronic kidney disease.

**Table 2:** Operation.

	RNU (*N* = 109)	PU (*N* = 48)	*P* value
Laparoscopy	79 (72.48%)	19 (39.58%)	<0.001
Blood transfusion	7 (6.4%)	2 (4.2%)	0.723
Operation time (mins)	288 (110–455)	141 (64–340)	<0.001
Length of stay (d)	21 (8–67)	17.5 (5–37)	0.045

RNU: radical nephroureterectomy; PU: partial ureterectomy.

**Table 3:** The follow-up results of renal outcomes.

	RNU	PU	*P* value
3 months (*n*)	109	48	
eGRF	59.81 ± 24.3	61.82 ± 25.78	0.64
CKD III or higher	58 (53.21%)	24 (50%)	0.711
12 months (*n*)	105	40	
eGRF	53.43 ± 22.58	62.78 ± 25.81	0.034
CKD III or higher	64 (58.72%)	17 (35.42%)	0.046
24 months (*n*)	92	33	
eGRF	50.01 ± 20.36	61.56 ± 25.68	0.010
CKD III or higher	58 (53.21%)	12 (25%)	0.008

RNU: radical nephroureterectomy; PU: partial ureterectomy; eGFR: estimated glomerular filtration rate; CKD: chronic kidney disease.

**Table 4:** Multivariate Cox regression analysis.

Variable	HR (95% CI)	*P* value
Age	1.035 (1.003–1.067)	0.032
Sex	1.876 (0.987–3.566)	0.055
CKD III or higher	0.260 (0.124–0.544)	<0.001
PU	0.534 (0.246–1.158)	0.122

CKD: chronic kidney disease; PU: partial ureterectomy.
